# Dust on the Underground

**Published:** 1920-10-09

**Authors:** 


### DUST ON THE UNDERGROUND.

That a great quantity of dust, paper, and other *débris* needs daily removal from the underground railways of London is an accepted fact, but it is more than probable that the existing system of removing such *débris* might well be dispensed with and another method adopted.

At various times in the day one meets on platforms, stairways, subways, or lifts, young underground officials who, armed with large brooms and boisterous spirits, endeavour with much vigour to remove all dust and untidyness, and we must say they do achieve their end, but at what cost? Clouds and clouds of dust and the creation of an atmosphere highly charged with irritating particles injurious alike to the nasal mucous membrane, the ocular conjunctiva, the bronchi, and the good temper of our long-suffering populace. It is a common experience to enter, at any time of the day, a lift which has just undergone its midday toilet, to meet an atmosphere gloomy with dust and a floor a swamp of moist dirty planking—the result of a very free sprinkling with a very odorous antiseptic. It is certainly to the credit of the Company that they use antiseptics for this purpose and supply them liberally to the employés; but the method of using them could be improved.

Such a state of affairs, together with the terrible daily crowding, must militate against the good health of both passengers and employés, and because of this we feel that a protest is justifiable, and, indeed, overdue.

To us it seems a reasonable suggestion that *all cleaning* of the tube passenger-ways, even into their most diverse ramifications, should be carried out in the small hours of the early morning, or other time when passengers are not travelling. But in any case, the present method is somewhat archaic when we realise that with electric power at hand the vacuum system might be employed by which all *débris* could easily be removed in a speedy and healthy fashion.

We realise that the finances of the underground railway companies are not in an ideal state, and that any innovation which means a heavy expenditure of money will be looked upon with marked disfavour. Yet the health of the people is of as great a consequence, and it is with this end in view, and this alone, that we bring the matter before the notice of our readers.

